# Editorial: Diagnosis, animal models and therapeutic interventions for neuromuscular diseases

**DOI:** 10.3389/fgene.2024.1481705

**Published:** 2024-09-11

**Authors:** Muthukumar Karuppasamy, Martine Tetreault, Jessica Rosati

**Affiliations:** ^1^ Department of Pediatrics, Division of Neurology at the University of Alabama at Birmingham and Children’s of Alabama, Birmingham, AL, United States; ^2^ University of Montreal Hospital Research Center (CRCHUM), Montreal, QC, Canada; ^3^ Department of Neuroscience, University of Montreal, Montreal, QC, Canada; ^4^ Cellular Reprogramming Unit, Fondazione IRCCS Casa Sollievo della Sofferenza, San Giovanni Rotondo, Italy

**Keywords:** neuromuscular diseases, musculoskeletal disorders, muscular dystrophy, animal models, drug repurposing/repositioning/rescue (DRPx)

Neuromuscular disorders (NMDs) are a group of rare diseases that largely affect the muscles, peripheral nervous system and neuromuscular junctions. Several genetic variations are associated with various types of NMDs. The fatal consequences of NMDs include skeletal muscle waste, reduction in muscle strength, muscle degeneration, neuropathy, spinal cord and neuromuscular junction abnormalities, sensory and motor impairment, lack of mobility and/or defects in heart and lungs (Miller, 2008; Vissing, 2024; Zambon et al., 2024). The clinical diagnosis and the clinical management of NMDs in children and adults is very challenging since the majority of NMDs patients present unspecific clinical and pathological features that are similar between NMD sutypes (Mary et al., 2018; Angelini, 2012). The current diagnostic approaches include genetic testing, biochemical evaluation, muscle and nerve biopsy, computerized tomography, electromyography and magnetic resonance imaging (Holm et al., 2022; Ravi et al., 2019; Dabaj et al., 2024; Rathore and Kang, 2023).

Technological progress has greatly improved our ability to diagnosed patients affected with NMDs; however, the majority are still very limited treatment options (Mackels and Servais, 2024). As an example, Duchenne muscular dystrophy patients have been treated with corticosteroids which exhibit detrimental adverse effects such as weight gain, behavioral problems and fragile bones. As for modeling of diseases, there are currently no efficient animal models to recapitulate key phenotypes seen in most NMDs patients. Absence of animal models further hinders the drug development for NMDs. Therefore, therapeutic strategies aimed at curing these NMDs efficiently are warranted now. The drug discovery and development approaches including gene editing, drug repurposing, RNA-based intervention and gene replacement therapy have greatly helped toward clinical trials and other drug developmental phases conducted for various NMDs. In the current scenario, creating novel animal models, gene therapy and biomarkers for NMDs have been on the rise as it may be beneficial in understanding disease pathology, evaluating variant-specific therapeutic interventions and advancing them into patient use. The key goal of this Research Topic is to report novel diagnosis, therapeutic interventions, animal models, disease management in NMDs. This Research Topic is also aimed at incorporating the newer diagnostic approach or genetic screening method for any NMDs. Multiple manuscripts were submitted for the publication of this Research Topic and a few articles were accepted and published.

Tang et al. presented a case carrying a variant in the *SCP2* gene (OMIM number: 184755), known for its involvement in the process of peroxisomal beta oxidation. To date, only two cases of SCPx deficiency have been reported. This patient has symmetrical lesions of the thalamus and brainstem, as previously described, but this variant causes a notably different clinical phenotype characterized by episodic psychosis. It may be suggested that homozygosity for a rare variant brings to question potential for consanguinity and other potential causes of an unusual phenotype. The authors identified a homozygous splicing variant resulting in skipping of exon 8 and the consequent loss of 29 amino acids in the SCP2 protein. In another study, Swindell conducted a meta-analysis of six lower motor neuron gene expression studies obtained by laser-capture microdissection of post-mortem spinal cord from ALS and control subjects. Of note, the six datasets included 56 ALS patients (with both sporadic and genetic ALS such as *C9ORF72*, *SOD1* and *CHMP2B*) and 37 healthy donors. This study identified 500 differentially expressed genes (DEGs) among ALS and CTL samples highlighting 10 key functional categories implicated in the disease. The analysis also connected ALS transcriptomics to genetics, first by demonstrating overlap between DEGs and genes near ALS-associated SNP loci, and second by identifying putative DNA regulatory elements disrupted or engendered by ALS-associated SNP variants. The author also compared these DEGs with DEGs from dissected motor neurons from both the SOD1-G93A mice and the iPSC-MNs. In the last model, most DEGs were not replicated. The outcome from this study could be beneficial in exploring the disease mechanisms and genetics underlying ALS. Wang et al. performed a systematic review and meta-analysis for diagnostic utility of long non-coding RNAs (lncRNAs) in multiple sclerosis (MS). The authors used the AUADAS-2 tool to test the selected studies from various Chinese and English literature databases. The meta-analysis included data from 12 articles and 24 studies. This study outlined the key role of lncRNAs in the diagnosis of MS, especially in distinguishing relapsing-remitting multiple sclerosis (RRMS) from healthy controls and across various MS subtypes. Thus, the authors demonstrate a critical role of lncRNAs not only in the diagnosis of MS, but also in predicting its progression and assessing disease severity suggesting that the integration of lncRNAs into clinical practice could significantly improve the management and stratification of MS patients, providing a basis for personalized therapeutic strategies.

Gao et al. reported a two sample Mendelian randomization study on the relationship of interferon-γ and interleukin-18 upstream of intervertebral disc degeneration (IVDD). They used publicly available inflammatory cytokines and IVDD GWAS data and demonstrated the casual relationship between IFN-γ and IL-18 and IVDD via instrumental variable analysis. This study could offer invaluable insights on IVDD monitoring, prevention and screening of drug targets.

We thank all the authors for their studies that are very useful to the NMDs scientific community as well as the general public. We hope that the novel findings published in this Research Topic will have a significant impact on the diagnosis, animal models development, and therapeutic interventions for NMDs.
